# Change of Optical Coherence Tomography Morphology and Associated Structural Outcome in Diabetic Macular Edema after Ranibizumab Treatment

**DOI:** 10.3390/jpm12040611

**Published:** 2022-04-11

**Authors:** Nan-Ni Chen, Chien-Hsiung Lai, Chai-Yi Lee, Chien-Neng Kuo, Ching-Lung Chen, Jou-Chen Huang, Pei-Chen Wu, Pei-Lun Wu, Chau-Yin Chen

**Affiliations:** 1 Department of Ophthalmology, Chang Gung Memorial Hospital, Chiayi 61363, Taiwan; nani4chen@gmail.com (N.-N.C.); jackeichen@gmail.com (C.-L.C.); peilun@cgmh.org.tw (P.-L.W.); ccy423@gmail.com (C.-Y.C.); 2 Department of Nursing, Chang Gung University of Science and Technology, Chiayi 61363, Taiwan; 3 School of Traditional Chinese Medicine, College of Medicine, Chang Gung University, Taoyuan 33302, Taiwan; 4 Department of Ophthalmology, Show Chwan Hospital, Changhua 50861, Taiwan; ao6u.3msn@hotmail.com; 5 Department of Ophthalmology, Changhua Christian Hospital, Changhua 50861, Taiwan; terrry339@yahoo.com.tw; 6 Department of Optometry, Chung Hwa University of Medical Technology, Taipei 10650, Taiwan; 7 Department of Ophthalmology, Taipei Medical University Hospital, Taipei 110301, Taiwan; roro691213@gmail.com; 8 Department of Ophthalmology, School of Medicine, College of Medicine, Taipei Medical University, Taipei 110301, Taiwan; 9 Department of Ophthalmology, Landseed International Hospital, Taoyuan 32449, Taiwan; heyshowjim77@gmail.com

**Keywords:** optical coherence tomography, diabetic macular edema, antivascular endothelial growth factors, vitreomacular interface abnormality

## Abstract

(1) Background: To investigate the correlation between therapeutic outcome and morphologic changes for diabetic macular edema (DME) after intravitreal injection of ranibizumab (IVIR). (2) Methods: This retrospective study included 228 eyes received IVIR for DME. Each participant was traced for two years after the initial IVIR, while the data of ophthalmic examination, optical coherence tomography (OCT) image, and systemic diseases were collected. The study population was categorized into different subgroups according to the existence of OCT morphologic change and the initial OCT morphologic pattern, including diffuse retinal thickening (DRT), cystoid macular edema (CME), serous retinal detachment (SRD), and vitreomacular interface abnormalities (VMIAs). The primary outcomes were the baseline best-corrected visual acuity (BCVA) and central macular thickness (CMT) during a two-year study period. The distribution of OCT morphologic change and its relation to primary outcome were analyzed. (3) Results: Comparing the 42 eyes (18.4%) with OCT morphological changes to another 186 eyes (81.6%) without such alteration, the former showed a poorer baseline BCVA (0.84 ± 0.39 vs. 0.71 ± 0.36, *p* = 0.035), worse final BCVA (0.99 ± 0.44 vs. 0.67 ± 0.30, *p* = 0.001), and thicker final CMT (354.21 ± 89.02 vs. 305.33 ± 83.05, *p* = 0.001). Moreover, the VMIA developed in 14.9% of all DME patients presenting the most common morphologic change among DRT, CME, and SRD. Besides, the presence of stroke was independently correlated to the morphologic change (adjusted odds ratio [aOR]: 6.381, 95% confidence interval (CI): 1.112–36.623, *p* = 0.038). (4) Conclusions: The change of OCT morphology in DME patients receiving IVIR was correlated to worse structural and visual outcome while the formation of VMIA most commonly occurred after initial treatment.

## 1. Introduction

Spectral domain optical coherence tomography (SD-OCT) has been rapidly developed and widely used for detection of alternation and quantification of retinal structure in various types of macular edema [1]. Several patterns of diabetic macular edema (DME) as well as OCT parameters have been proposed on SD-OCT, including diffuse retinal thickening (DRT), cystoid macular edema (CME), serous retinal detachment (SRD), and vitreomacular interface abnormalities (VMIAs) [1,2,3,4,5,6,7,8].

As intravitreous anti-vascular endothelial growth factors (anti-VEGF) injections have become the first-line therapy for DME [9,10], OCT patterns used in monitoring the effect of therapies for macular edema contribute to understanding the retinal anatomical response and structure damage of DME with distinctive aspects in each morphologic subtype [8]. Previous studies have reported that the effect of intravitreal anti-VEGF treatment is predictable among different patterns of DME [3,5,6,7]; however, seldom studies demonstrated the changes in OCT parameters over time after anti-VEGF therapy [11,12]. Although certain OCT morphology may be likely developed after anti-VEGF treatment for DME, the exact types need further validation.

Therefore, we aimed to investigate the association among different morphologic subtypes of DME and the response and changes in OCT morphological features over time after intravitreal ranibizumab (IVIR) injections.

## 2. Materials and Methods

### 2.1. Subject Selection 

The retrospective cohort study was conducted and the medical records of 652 eyes were reviewed from 433 patients with DME receiving at least one IVIR with 0.5 mg of ranibizumab (0.05 mL) during February 2013 and June 2019 in Chang Gung Memorial Hospital, Chiayi branch in southern Taiwan. The inclusion criteria included (1) clinically significant macular edema (CSME) as defined by ETDRS; and (2) CMT of >300 μm as documented on OCT. The exclusion criteria were as follows: (1) any other ophthalmic disease apart from diabetic retinopathy and cataract; (2) previous intravitreal bevacizumab, intravitreal aflibercept, or intravitreal steroid injections; (3) previous macular focal laser therapy; (4) intraocular surgery or panretinal photocoagulation within 3 months of commencement of the study period; and (5) any missing data or poor OCT image quality at designated time points. After the selection process, a total of 228 eyes of 150 patients with OCT scans comprised the study population for evaluation of OCT morphologic characteristics.

### 2.2. Data Collection

At first visit, all patients underwent complete ophthalmic examination, including best-corrected Snellen visual acuity (BCVA), slit-lamp examination, intraocular pressure measurement, fundus photography, retina thickness measurement by OCT, and fluorescence angiography. BCVA values were converted to the logarithm of the minimum angle of resolution (logMAR) scale for statistical manipulation. Metabolic parameters were also assessed through review of the comprehensive medical records, including hypertension (HTN), coronary artery disease (CAD), stroke, chronic kidney disease (CKD), hyperlipidemia, thyroid disease, and cancer. After the IVIR, patients were examined monthly until 3 months, then 6, 12, and 24 months for collecting the OCT morphology parameters. The decision to perform reinjection was based on persistent or increased sub-retinal fluid/cystoid macular edema, or CMT > 300 μm on OCT at the monthly control visits. If any morphology change occurred, it would be recorded at all designated time points.

### 2.3. Morphology Measurement

One SD-OCT device (Optovue Inc., Fremont, CA, USA) with software version 6.2.2.73 was applied for the image collection and analysis. The eyes were classified into 4 subgroups based on the cross-sectional retinal morphologies. The anatomic outcome was defined as CMT reduction between the baseline and the control visit. The CMT was automatically calculated as the average retinal thickness within the central circle of a 500-μm radius. At the designated period of time of 2 years, all patients were divided into two groups according to the presence of morphology changes. Patients with any OCT morphology changes were categorized as group 1 (42 eyes), while those without changes of OCT pattern were regarded as group 2 (186 eyes). The OCT patterns were classified into four types: (1) the DRT pattern characterized by a generalized, heterogeneous, sponge-like swelling of the macula with mild hyporeflectivity compared with normal retina; (2) the CME pattern defined by intraretinal oval cystoid spaces of low reflectivity, typically separated by highly reflective septae; (3) the SRD pattern showing a hyporeflective, dome-shaped detachment between the retinal pigment epithelium (RPE) and the retina; and (4)VMIA including the presence of epiretinal membranes (ERM) or vitreomacular traction (VMT). Both ERM and VMT presented as a highly reflective band or membrane on the retinal surface. All patients were divided into four groups based on the OCT findings, as our previous work have mentioned [13]. DRT group included patients with only pure DRT, while CME group included patients with CME, but no subretinal fluid or vitreous macular interface abnormalities. If DRT and CME coexisted, then CME was considered the dominant pattern. SRD group comprised patients with SRD, but without VMIA. When DRT, CME, and SRD all presented, the eye was classified into SRD group. Regardless of pattern combinations, eyes with ERM or vitreomacular traction were classified into VMIA group.

### 2.4. Statistical Analysis 

All analyses were computed by using the PASW Statistics 18 software (Version 18.0. SPSS Inc., Chicago, IL, USA). Descriptive analysis was used to show the baseline characteristics of the study group. Then, the basic properties as well as the changes of CMT between group 1 and group 2 were analyzed by using independent *t*-test and Chi-square test, respectively. Next, the rate of morphological changes among different OCT subgroups was analyzed by one-way analysis of variance with post-hoc exam of Dunnett T3. The bar chart was plotted to demonstrate the distribution of OCT morphology change among the different subgroups. Besides, the multivariate logistic regression analysis was utilized to yield adjusted odds ratio (aOR), corresponding 95% confidence interval (CI) for evaluating the correlation between OCT morphology alteration and demographic data as well as systemic parameters. A *p* value of less than 0.05 is regarded as statistical significance.

## 3. Results

### 3.1. Baseline Characteristics and Treatment Outcome

The baseline characteristics of the study population are summarized in Table 1. The mean age was 66.30 ± 9.47 years in the group 1 and 65.66 ± 9.01 in group 2 without significant difference (*p* = 0.720). The overall average dosages of injections for all patients were 4.31 ± 1.26 in the first year and 5.33 ± 1.90 in two years. Besides, no differences were noted between group 1 and group 2 regarding the baseline characteristics of gender, diabetes retinopathy stage (PDR/NPDR), panretinal photocoagulation, baseline CMT and average one-year and two-year injection dosage (all *p* > 0.05). However, the baseline BCVA, final BCVA, and CMT at second year of treatment were significantly different between two groups (*p* = 0.035, 0.001, and 0.001, respectively) in which group 1 was correlated to a poorer baseline and final BCVA (0.84 ± 0.39 and 0.99 ± 0.44, log MAR), and greater second year CMT (354.21 ± 89.02) (Table 1).

### 3.2. Subgroup Analysis for Morphological Changes

**Figure 1:**
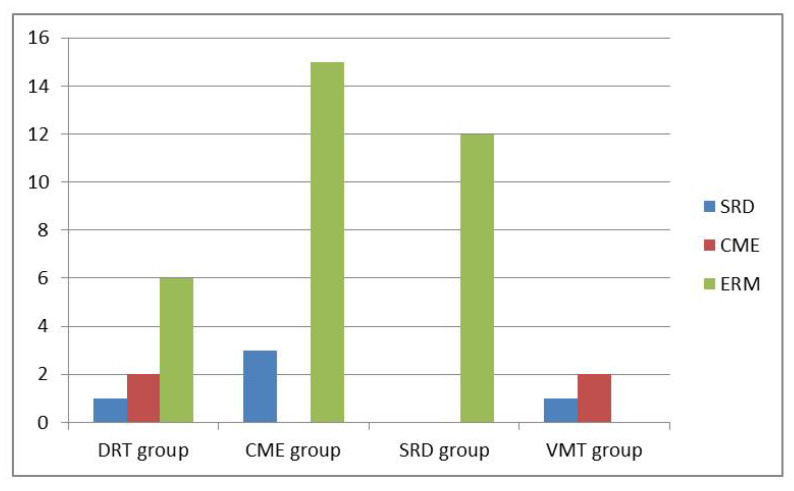
Distributions of morphological changes over the course of Ranibizumab treatment among different subtypes.

In the subgroup analysis, 38 eyes were classified into DRT group, 80 in CME group, 45 in SRD group, and 62 in VMIA group. All combinations of morphological subtypes of DME and number of conversions into another subtypes over time period of two years are summarized in Table 2. The percentage of OCT morphology changes among DRT, CME and SRD groups showed no significant difference (all *p* > 0.05). The distributions of morphological changes over the course of Ranibizumab treatment among different subtypes are shown in Figure 1. Moreover, VMIA was the most common type of change and developed in 14.9% of patients with DME receiving IVIR treatment during a follow-up period of 24 months. The number of clinically detectable ERM cases did not change significantly after the 2nd year with a mean period of 45.7 months follow-up. Overall, we found a mean period of 12.3 months when the first detection of changes between different DME subtypes.

### 3.3. Correlation between Systemic Parameters and Morphology

Regarding the potential confounding factors for the morphological change in DME, stroke was significantly associated with higher risk for developing into another DME subtypes (aOR: 6.381, 95% CI: 1.112–36.623, *p* = 0.038) using multivariate logistic regression analysis. However, other demographic data and systemic parameters did not reveal significant correlation to the change in OCT morphology (all *p* > 0.05) (Table 3).

## 4. Discussion

In DME, liquid accumulation can occur in intracellular or extracellular spaces due to cytotoxic or vasogenic process [14]. Furthermore, pathologic fibrocellular changes at the vitreomacular interface are present in eyes with DME irrespective of the type of macular edema as classified by SD-OCT. Several patterns of macular edema depending on the location of intracellular or extracellular fluid have been first described by Otani et al. [15]. Investigators have found variable results for visual and anatomic improvement in different groups after anti-VEGF treatment [4,5,6,7,16,17,18,19,20,21,22]. Similarly to our current study, changes in OCT parameters over time after IVIR treatment were noted. Compared with group 2 without morphology changes, group 1 showed a significant higher CMT at the second year (*p* = 0.001), indicating poor response to anti-VEGF therapy and other different key mechanisms involved in the development of DME. This finding suggests that the possible strategy may shift to dexamethasone therapy or even surgical treatment.

On the aspect of VMIA groups, any alterations of OCT morphology, such as newly developed CME, SRD, or macula contracture in the development and progression of DME could occur with previous exiting vitreomacular interface abnormality. One eye with ERM further developed macula pucker at the 12th month. The CMT outcome at the second year follow-up showed an average of 390.5 ± 75.3 µm in VMIA group patients with morphological changes. The finding had considerable discrepancy in our previous work, which had favorable improvement in reduction of CMT in VMIA group with consistent IVIR injections [13]. Patients with poor response, underlying physical mechanisms of pathogenesis other than VEGF, may be involved and receive benefit from the treatment of surgical relief of traction [23]. Nevertheless, among the eyes developing ERM during the anti-VEGF treatment course, the CMT outcome at the second year showed a better resolution of 19.33 µm with shorter follow-up period compared with VMIA group, though there was no statistically significant (*p* = 0.62). We still assume that this difference may be caused by distinct mechanisms of ERM formation. Previous studies have stated that anti-VEGF agents may be involved in the fibrotic process of diabetic eyes, induced by the intravitreal injection method or angio-fibrotic switch of VEGF and CTGF in PDR [24]. Besides, long-term intravitreal injection attributing to repetition of volume expansion may accelerate posterior vitreous detachment, which is beneficial in the natural history of DME [25]. The ERM associated with posterior vitreous detachment may instead provide a tension force toward the retina to prevent macula edema from progression in patients whose epiretinal membranes initiated secondary to the vasculopathy process at macula, and patients with repetition of intravitreal injections. Most importantly, though CMT could achieve favorable results, symptoms of retinal distortion resulting from ERMs are still considered as a surgical indication.

**Figure 2:**
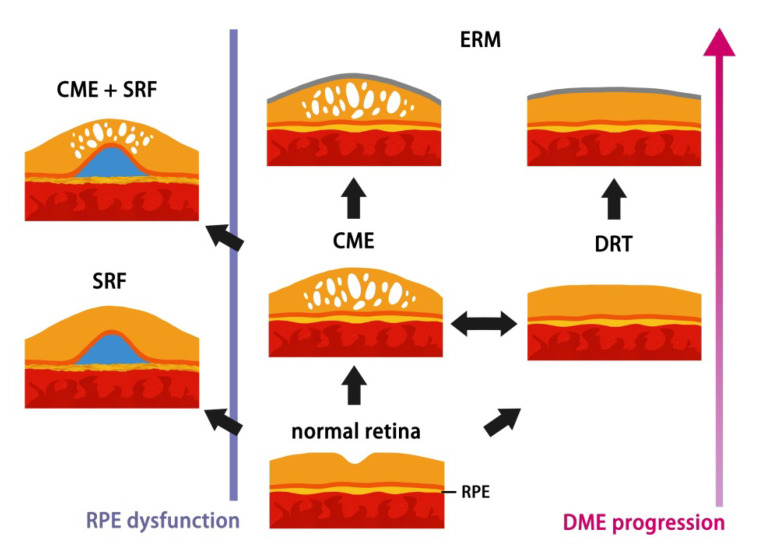
Schematic diagram of hypothesis of DME morphological procession changes on OCT.

Among three subtypes of DME, patients with CME or DRT had higher risk of development of VMIA than those with SRD. In accordance with the immunostaining result conducted by Haguenau, F. et al., which eyes with DRT and CME were most intensely positive for active myofibroblasts and the enzyme matrix metalloproteinase-9 (MMP-9) [26], our findings address the tendency for progression to proliferative disease in DRT and CME groups. In the present study, a succession of change was noticed from DRT to CME/SRD and from CME to SRD, although vasogenic or inflammatory etiology of DME is very difficult to distinguish from OCT. In contrary, none of eyes with pure SRD transformed into CME type after IVIR treatment, except the coexistence of initial VMIA. As vasogenic changes secondary to hyperglycemia begins the cascade of macular edema formation causing a failure in the RPE pump function [14,27,28], we suppose SRD may be an indicator of earlier stage DME, which responds well to IVIR and seldom transforms into other subtypes under intravitreal anti-VEGF therapy. Apparently, this finding correlates well with previous studies, which the presence of subretinal fluid has better response to treatment [11,29]. The individual differences of RPE function contributing to the presence of this early sign, provide in-depth explanation why part of patients developed SRD with longer DME duration and disease progression, as the schematic diagram illustrated in Figure 2.

As DM progressed, the hyperglycemia status initiates breakdown of RPE barrier. RPE cells could decompensate at any stages of DME on interindividual variation and then patients develop SRD. Consequently, accumulation of the fluid causes edema (CME/DRT). Fibrocellular proliferation on the internal limiting membrane further results in ERM secondary to DM retinopathy or intravitreal injections.

On the aspect of the association between DM retinopathy and cardiovascular diseases such as stroke or CAD, prior studies have addressed that patients with type 2 diabetes and DME or PDR have an increased risk of cardiovascular events [30]. In our present study, we found DME patient with stroke had a 6.4-fold increase in the relative risk of developing into another DME subtypes. This finding may account for the relationship between stroke and severe diabetes, or the relevant confounding factors related to stroke. Moreover, for patients taking aspirin regularly, the anti-inflammatory agent as a confounding factor may block the gap junction communication between the RPE cells, damage retinal microenvironment [31], and possibly worsen the progression of DME.

Our study has several limitations to be noted. First, this nonrandomized and uncontrolled study retrospectively collected data from a relatively small number of patients without standardized treatment regimen. Second, due to the reimbursement limitations, most patients were undertreated with a mean number of 5.33 injections over 24 months. Data were derived from poor-quality images, previous treatments, or short follow-up period resulted in relative large number of excluded cases. Yet, eyes with different duration of DME, control of DM, and timing for injections were not evaluated, reflecting real-life conditions in clinical practice.

## 5. Conclusions

In summary, macula edema disappeared rapidly after IVIR treatment and might recur or even evolve to other subtypes over time, indicating a disease progression with poor structure and visual outcome. Pathologic fibrocellular changes at the vitreomacular interface were the most common type of changes and presented in all eyes with DME regardless of the type of macular edema as classified by SD-OCT. We suppose SRD may be an indicator of earlier stage DME, which responds well to IVIR and seldom transforms into other subtypes under intravitreal anti-VEGF therapy. Future research should focus on exploring different etiologies in DME, which would allow clinicians to offer more precise and individualized treatments.

## Figures and Tables

**Table 1:** Demographic and clinical properties of the patients.

Description	Group 1	Group 2	*p*-Value
No. of eyes	42	186	
Male/Female	19/14	76/41	0.437
Age, years	66.30 ± 9.47	65.66 ± 9.01	0.720
Baseline BCVA (logMAR)	0.84 ± 0.39	0.71 ± 0.36	0.035
2nd year BCVA (logMAR)	0.99 ± 0.44	0.67 ± 0.30	0.001
Baseline CMT	422.07 ± 151.11	412.16 ± 109.67	0.690
2nd year CMT	354.21 ± 89.02	305.33 ± 83.05	0.001
1 year dosage	4.40 ± 1.13	4.30 ± 1.31	0.618
2 years dosage	5.43 ± 1.71	5.31 ± 1.95	0.709
HbA1c	7.56 ± 1.09	7.56 ± 1.19	0.993
NPDR/PDR	26/16	114/72	0.941
PRP	33	130	0.260
Pseudophakia	9	51	0.426
High Myopia	2	5	0.616
Smoking	1	6	1.000
Hypertension	23	70	0.302
Hyperlipidemia	4	23	0.320
Coronary artery disease	2	7	1.000
Stroke	4	5	0.108
Chronic kidney disease	9	21	0.237
Thyroid disease	0	1	1.000
Cancer	2	4	0.613

Group 1: patients with presence of OCT morphological changes after treatment; Group 2: patients without OCT morphological changes after treatment; BCVA: best corrected visual acuity; CMT: central macula thickness; PDR: proliferative diabetic retinopathy; NPDR: non-proliferative diabetic retinopathy; PRP: panretinal photocoagulation.

**Table 2:** Combinations of morphological subtypes and changes over time period of 2 years.

OCT Patterns	All No. (%)	No. of Changes over Time Period of 2 Years (%)
DRT group		
DRT alone	38 (16.67%)	9 (23.7%)
Combined pattern	0 (0%)	0 (0%)
Total	38 (16.67%)	9 (23.7%)
CME group		
CME alone	20 (8.77%)	3 (15.0%)
Combined pattern	60 (26.3%)	14 (23.3%)
Total	80 (35.09%)	17 (21.3%)
SRD group		
SRD alone	7 (3.07%)	0 (0%)
Combined pattern	38 (16.67%)	11 (28.95%)
Total	45 (19.7%)	11(24.44%)
VMIA group		
ERM alone	13 (5.70%)	1 (7.7%)
Combined pattern	52 (22.80%)	3 (5.77%)
Total	65 (28.5%)	4 (6.15%)

**Table 3:** Multivariate logistic regression analysis of morphological changes.

Variable	S.E	OR (95% CI)	*p*-Value
Intercept	1.614		0.117
Age	0.024	1.012 (0.966, 1.060)	0.615
Gender	0.440	1.425 (0.602, 3.375)	0.421
Smoking	1.242	0.487 (0.043, 5.555)	0.563
Alcohol	0.963	1.743 (0.264, 11.500)	0.564
HbA1c	0.186	1.061 (0.737, 1.526)	0.750
Hypertension	0.460	1.459 (0.593, 3.593)	0.411
CAD	0.933	0.631 (0.101, 3.925)	0.622
Stroke	0.892	6.381 (1.112, 36.623)	0.038
CKD	0.525	2.316 (0.828, 6.476)	0.109
Cancer	0.931	1.085 (0.175, 6.733)	0.930
Hyperlipidemia	0.728	0.258 (0.062, 1.075)	0.063

CAD: coronary artery disease; CKD: chronic kidney disease.

## Data Availability

The data related to the current study is available upon reasonable request from the editorial board.
